# Telehealth use and perceptions among prostate cancer survivors

**DOI:** 10.1002/cam4.6328

**Published:** 2023-07-16

**Authors:** Luke W. Chen, Deborah S. Usinger, Aaron J. Katz

**Affiliations:** ^1^ Department of Radiation Oncology University of Kansas Cancer Center Kansas City Kansas USA; ^2^ Lineberger Comprehensive Cancer Center University of North Carolina at Chapel Hill Chapel Hill North Carolina USA; ^3^ Department of Population Health University of Kansas Medical Center Kansas City Kansas USA

**Keywords:** cancer survivors, sociodemographic factors, telemedicine

## Abstract

**Background:**

Reasons underlying disparities in telehealth use among cancer survivors are unknown.

**Methods:**

We surveyed a sociodemographically diverse population‐based cohort of 487 prostate cancer survivors regarding their use and perceptions of telehealth during the COVID‐19 pandemic.

**Results:**

Overall, only 28.5% of survivors had used telehealth at the time of survey and just 10% felt care through telehealth is comparable to that of an in‐person visit. Still, over 55% felt telehealth is a good option for initial consultations or basic care and 15% felt more likely to use telehealth since the pandemic. After adjusting for other socioeconomic factors, survivors with lower education (≤high school vs. any college) had marginally lower use of telehealth (risk ratio [RR], 0.65 [95% CI, 0.42–1.01]) and lower probability of feeling more likely to use telehealth since the pandemic (RR, 0.39 [95% CI, 0.20–0.77]).

**Conclusions:**

Differences in survivor perceptions of telehealth by education level highlight new insights underlying disparities in telehealth use and potential targets for interventions.

## 1 INTRODUCTION

Telehealth, which comprises clinical visits provided through phone or video calls, has grown rapidly in the Unites States since the start of the COVID‐19 pandemic. Providers may leverage telehealth to consult and deliver care to patients in conjunction with traditional in‐person visits and/or when in‐person care is not feasible or readily available. Studies have shown that telehealth can benefit cancer survivors by increasing their access to specialists and health care services,^1^, ^2^ and by improving patient outcomes such as physical functioning and quality of life.^3^ Nevertheless, prior research has also demonstrated important differences in patient uptake of telehealth by patient age, sex, race/ethnicity, rurality, and measures of socioeconomic status (SES).^4^, ^5^, ^6^, ^7^, ^8^

Among cancer survivors, variation in telehealth use by social and economic patient characteristics may contribute to or exacerbate disparities, particularly if such variation equates to differences in access to cancer specialists or care. However, earlier studies demonstrating differences in telehealth uptake among cancer survivors are limited by their use of data from single academic institutions, reliance on area‐level composite measures of SES, and/or lack of information on patient perceptions of telehealth, which may influence its use. To better understand barriers that may contribute to differences in telehealth use among cancer survivors, further studies are needed to examine patient‐level SES factors, along with patient perceptions of telehealth. We assess this in a prospectively followed, population‐based cohort of long‐term prostate cancer survivors.

## 2 METHODS

The North Carolina Prostate Cancer Comparative Effectiveness & Survivorship Study (NC ProCESS) is a population‐based, prospective cohort study of prostate cancer survivors. Details about the study design of NC ProCESS have been reported previously.^9^ Briefly, from 2011 to 2013, men newly diagnosed with localized prostate cancer were identified through the Rapid Case Ascertainment system of the North Carolina Central Cancer Registry from across all 100 counties of the state. All study participants signed written informed consent and were enrolled prior to treatment and followed prospectively on an annual basis using telephone surveys. During annual follow‐up surveys between December 2020 and September 2022, participants were asked specific questions about their use and perceptions of telehealth (Table S1).

Descriptive statistics were used to summarize results, and multivariable Poisson regression models assessed potential associations between sociodemographic factors and telehealth use and perceptions. All analyses were performed using SAS, version 9.4 (SAS Institute; Cary, NC).

## 3 RESULTS

Of the 759 prostate cancer survivors we attempted to contact, 537 (70.8%) were successfully reached and 487 (64.2%) completed the survey. The 272 survivors who did not complete the survey were more likely to have a household income ≤$40,000 (51.1%) than those who completed the survey; no other notable differences were identified.

This sociodemographically diverse cohort had a mean (SD) age of 73.0 (7.3) years; 25.1% were Black, 28.8% had ≤ a high school education, 33.5% had a household income ≤ $40,000, and 44.1% reported living in a rural setting. Of the 487 survivors, 57.3%, 31.6%, and 11.1% had low‐risk, intermediate‐risk, and high‐risk prostate cancer at the time of their initial diagnosis. Approximately 90% reported that their prostate cancer had never come back or progressed; only 12 survivors (2.5%) reported receiving treatment within 12 months of completing the survey. A total of 139 (28.5%) survivors had used telehealth at the time of survey. The percentage who used telehealth was greater among survivors who were < 65 years old, Black, attained any college education, and lived in an urban or mixed setting (Table 1). Only 10% of survivors felt care through telehealth is comparable to that of an in‐person visit, but over 55% felt telehealth is a good option for initial consultations or basic care. Overall, 16.4% of survivors felt they were more likely to use telehealth since the pandemic while 12.3% reported feeling less likely (Table S2); however, there were noticeable differences by survivor education level. Approximately 20% of survivors who attained any level of college felt more likely to use telehealth since the pandemic compared to just 8% with a high school education or less.

**TABLE 1:** Consideration for and perceptions about telehealth among prostate cancer survivors, by patient subgroups.

		Considered telehealth and had a telehealth appointment	Feel that care through telehealth is comparable to that of an in‐person visit	Feel that care through telehealth is not comparable to an in‐person visit, but it is a good option for initial consultations and/or basic care	Are more likely to use telehealth since the COVID‐19 pandemic
Characteristic	*N*	*n* (%)	*n* (%)	*n* (%)	*n* (%)
All participants	487	139 (28.5)	49 (10.1)	273 (56.1)	80 (16.4)
Age					
<65 years	72	27 (37.5)	10 (13.9)	40 (55.6)	16 (22.2)
≥65 years	415	112 (27.0)	39 (9.4)	233 (56.1)	64 (15.4)
Race					
Black	122	42 (34.4)	18 (14.8)	56 (45.9)	24 (19.7)
White	351	94 (26.8)	30 (8.5)	208 (59.3)*	53 (15.1)
Other	14	3 (21.4)	1 (7.1)	9 (64.3)	3 (21.4)
Education					
≤high school	140	29 (20.7)	13 (9.3)	66 (47.1)	11 (7.9)
Any college	347	110 (31.7)*	36 (10.4)	207 (59.7)*	69 (19.9)**
Household income					
≤$40,000	163	42 (25.8)	15 (9.2)	78 (47.9)	20 (12.3)
>$40,000	313	93 (29.7)	34 (10.9)	188 (60.1)*	59 (18.8)
Rural–urban residence^a^					
Rural	224	53 (23.7)	17 (7.6)	124 (55.4)	29 (12.9)
Urban/Mixed	261	86 (33.0)*	32 (12.3)	148 (56.7)	51 (19.5)
Time of survey					
December 2020 to December 2021	344	92 (26.7)	37 (10.8)	195 (56.7)	55 (16.0)
January 2022 to November 2022	143	47 (32.9)	12 (8.4)	78 (54.5)	25 (17.5)

^a^ Participants reported whether they considered themselves to live in a primarily rural, primarily urban or mixed location at the time of the survey.

* *p* < 0.05

** *p* < 0.01.

On multivariable analysis (Table 2), lower education level (≤high school vs. any college) was marginally associated with less telehealth use (risk ratio [RR], 0.65 [95% CI, 0.42–1.01]) and a decreased probability of feeling more likely to use telehealth since the pandemic (RR, 0.39 [95% CI, 0.20–0.77]) after adjusting for other SES factors. Rural (vs. urban/mixed) residence was associated with lower telehealth use among survivors surveyed between December 2020 and December 2021 (RR, 0.62 [95% CI, 0.40–0.96]), but not among those surveyed in January–November 2022 (RR, 1.16 [95% CI, 0.64–2.12]) or overall (RR, 0.76 [95% CI, 0.54–1.08]).

**TABLE 2:** Association of prostate cancer survivor characteristics with use of telehealth and perceptions about telehealth during the COVID‐19 pandemic^a^.

	Considered telehealth and had a telehealth appointment	Feel that care through telehealth is comparable to that of an in‐person visit	Feel that care through telehealth is either “comparable to that of an in‐person visit” or “a good option for initial consultations and/or basic care”	Are more likely to use telehealth since the COVID‐19 pandemic
Characteristic	Risk ratio (95% CI)	Risk ratio (95% CI)	Risk ratio (95% CI)	Risk ratio (95% CI)
Age				
<65 years vs. ≥65 years	1.36 (0.88, 2.09)	1.40 (0.69, 2.85)	1.09 (0.80, 1.49)	1.42 (0.81, 2.48)
Race				
Black vs. White	1.33 (0.90, 1.96)	1.81 (0.97, 3.38)	0.95 (0.72, 1.25)	1.57 (0.94, 2.61)
Other vs. White	0.95 (0.29, 3.09)	1.12 (0.15, 8.51)	1.19 (0.60, 2.37)	2.30 (0.68, 7.82)
Education				
≤high school vs. any college	0.65 (0.42, 1.01)	0.88 (0.44, 1.76)	0.86 (0.65, 1.13)	0.39 (0.20, 0.77)
Household income				
≤$40,000 vs. >$40,000	0.95 (0.63, 1.41)	0.78 (0.40, 1.52)	0.85 (065, 1.10)	0.73 (0.42, 1.27)
Rural–urban residence^b^				
Rural vs. urban/mixed	0.77 (0.54, 1.09)	0.64 (0.35, 1.17)	0.94 (0.75, 1.17)	0.76 (0.48, 1.21)
Time of survey				
January 2022 to November 2022 vs. December 2020 to December 2021	1.19 (0.83, 1.69)	0.74 (0.39, 1.43)	0.92 (0.72, 1.17)	1.01 (0.63, 1.64)

Abbreviation: CI, confidence interval.

^a^ Model controlled for participant age group, race, education, income, rurality, and time of survey.

^b^ Participants reported whether they considered themselves to live in a primarily rural, primarily urban or mixed location at the time of the survey.

## 4 DISCUSSION

This study found differential uptake of telehealth by education level among long‐term prostate cancer survivors, aligning with previous reports of disparities in telehealth use during the COVID‐19 pandemic; several prior studies advocated for further research to elucidate reasons and barriers underlying this disparity.^4^, ^5^, ^10^, ^11^ The current investigation is one of few studies to examine perceptions of telehealth in a large cohort of cancer survivors and provides some new insights: First, only one in 10 prostate cancer survivors felt telehealth is comparable to an in‐person visit, but over 50% felt telehealth is a good option for initial consultation or basic care. Second, survivor consideration for and perceived likelihood of using telehealth differed significantly by education level.

One potential explanation for differential telehealth uptake relates to internet and technology access and comfort. In a study by Lama et al., rural versus urban individuals reported lower telehealth availability.^12^ Another likely barrier involves patient perceptions about telehealth. In a study of semi‐structured interviews with 20 cancer patients, patient‐noted downsides of telehealth included provision of less thorough care and issues with technology access.^13^ In a survey of 539 cancer survivors, Arem et al reported that only about one in four felt telehealth is appropriate for a new consultation^1^; in contrast, nearly 60% felt telehealth was appropriate for management of symptoms and for discussion of imaging or laboratory results. Results from the current study are consistent with these findings and provide insights concerning variation in perceptions of telehealth across socioeconomic characteristics of cancer survivors—which may help explain the disparities in telehealth uptake reported by prior studies. Although not statistically significant, the percentage of survivors who felt telehealth is a good option for initial consultations or basic care was notably smaller among those who attained a high school education or less, aligning with the lower telehealth use and lower perceived likelihood of using telehealth observed in this group.

Strengths of this study include its population‐based design, which provides more generalizable results than single‐institutional studies, and the diversity of participants, including almost half from rural areas. This study also represents one of the largest to date in assessing associations between SES factors and telehealth uptake among cancer survivors. Limitations include results from a single state and the absence of validated surveys on perceptions of telehealth for use in this study. In addition, we did not collect information on perceptions of telehealth related to specific types of visits or providers; cancer survivor views of telehealth may vary depending on the type of appointment and care services being provided.^1^ Lastly, the study was performed among a cohort of long‐term, localized prostate cancer survivors whose experiences and perceptions about telehealth may be distinct from other patient populations, including those newly diagnosed and/or with other types of cancer.

Telehealth increases access to care and has the potential to reduce cancer disparities, but most cancer survivors do not believe that telehealth can fully replace in‐person visits. Differences in survivor perceptions of telehealth by education level provide insights on disparities in telehealth uptake during the COVID‐19 pandemic and a potential target for interventions to reduce these differences.

## AUTHOR CONTRIBUTIONS

**Luke W Chen:** Conceptualization (equal); writing – original draft (equal); writing – review and editing (equal). **Deborah S Usinger:** Data curation (equal); project administration (equal); writing – review and editing (equal). **Aaron J. Katz:** Conceptualization (equal); data curation (equal); formal analysis (equal); methodology (equal); supervision (equal); writing – original draft (equal); writing – review and editing (equal).

## FUNDING INFORMATION

This work was supported by the Agency for Healthcare Research and Quality, U.S. Department of Health and Human Services as part of the DEcIDE program, contract HHSA290‐2005‐0040‐ITO6; and the Department of Defense U.S. Army Medical Research Acquisition Activity, Prostate Cancer Research Program (Award # W81XWH‐19‐1‐0512). The funders had no role in the design of the study; the collection, analysis, and interpretation of the data; the writing of the manuscript; or the decision to submit the manuscript for publication.

## CONFLICT OF INTEREST STATEMENT

The authors declare no conflict of interest for this study.

## ETHICS STATEMENT

This study was approved by the Institutional Review Board of the University of North Carolina at Chapel Hill.

## Supporting information


Table S1.
Table S2.

## Data Availability

Anonymized study data underlying this article can be made available on reasonable request to the corresponding author.
