# Supplementary material for: Telehealth use and perceptions among prostate cancer survivors
Source: Cancer Med. 2023 Jul 16;12(16):17308–12. doi: 10.1002/cam4.6328 (PMC10501258; doi:10.1002/cam4.6328)
Supplement: Supplementary file 1 — Table S1. Table S2. [file CAM4-12-17308-s001.docx]

**Supplementary Materials**

Supplement to: Chen LW, et al. Telehealth use and perceptions among prostate cancer survivors

**Supplementary Table 1.** Questions asked of prostate cancer survivors to assess their telehealth use and perceptions during the COVID-19 pandemic

**Supplementary Table 2.** Prostate cancer survivors' level of consideration for and perceptions of telehealth during the COVID-19 pandemic

**Supplementary Table 1. Questions asked of prostate cancer survivors to assess their telehealth use and perceptions during the COVID-19 pandemic**

| **Question** | **Response options** |
| --- | --- |
| “Have you considered trying a telehealth appointment?” | 1. Yes, I have already had a telehealth appointment  2. Yes, I have considered it, but I haven’t yet had a telehealth appointment  3. No, but I would consider a telehealth appointment  4. No, and I would never consider a telehealth appointment  5. Don’t know/not sure |
| “Do you feel that people get comparable health care through telehealth as they do for in-person visits?” | 1. Yes, I think the care is comparable  2. No but telehealth is a good option for initial consultations and/or basic care  3. No, telehealth care will never match the quality of an in-person visit  4. Don’t know/not sure |
| “Has the COVID-19 pandemic changed your view of telehealth?” | 1. I am more likely to use telehealth since the COVID-19 pandemic  2. I have the same opinion compared to before the COVID-19 pandemic  3. I am less likely to use telehealth since the COVID-19 pandemic  4. Don’t know/not sure |

**Supplementary Table 2.** Prostate cancer survivors' level of consideration for and perceptions of telehealth during the COVID-19 pandemic, overall and by study participant subgroups

|  |  | **“Have you considered trying a telehealth appointment?”** | | | |
| --- | --- | --- | --- | --- | --- |
|  |  | **Yes, considered telehealth and had a telehealth appointment** | **Yes, considered telehealth, but haven’t yet had a telehealth appointment** | **No, but would consider a telehealth appointment** | **No, and would never consider a telehealth appointment** |
| **Characteristic** | **N** | **n (%)** | **n (%)** | **n (%)** | **n (%)** |
| **All participants** | 487 | 139 (28.5) | 59 (12.1) | 191 (39.2) | 94 (19.3) |
| **Age** |  |  |  |  |  |
| <65 years | 72 | 27 (37.5) | 9 (12.5) | 29 (40.3) | 7 (9.7) |
| ≥65 years | 415 | 112 (27.0) | 50 (12.0) | 162 39.0) | 87 (21.0) |
| **Race** |  |  |  |  |  |
| Black | 122 | 42 (34.4) | 16 (13.1) | 39 (32.0) | 24 (19.7) |
| White | 351 | 94 (26.8) | 41 (11.7) | 147 (41.9) | 66 (18.8) |
| Other | 14 | 3 (21.4) | 2 (14.3) | 5 (35.7) | 4 (28.6) |
| **Education** |  |  |  |  |  |
| ≤high school | 140 | 29 (20.7) | 21 (15.0) | 50 (35.7) | 39 (27.9) |
| Any college | 347 | 110 (31.7)* | 38 (11.0) | 141 (40.6) | 55 (15.9)** |
| **Household income** |  |  |  |  |  |
| ≤$40,000 | 163 | 42 (25.8) | 24 (14.7) | 52 (31.9) | 43 (26.4) |
| >$40,000 | 313 | 93 (29.7) | 34 (10.9) | 136 (43.5)* | 48 (15.3)** |
| **Rural-urban residence^a^** |  |  |  |  |  |
| Rural | 224 | 53 (23.7) | 26 (11.6) | 91 (40.6) | 52 (23.2) |
| Urban/Mixed | 261 | 86 (33.0)* | 33 (12.6) | 99 (37.9) | 42 (16.1)* |
| **Time of survey** |  |  |  |  |  |
| Dec 2020 – Dec 2021 | 344 | 92 (26.7) | 38 (11.0) | 151 (43.9) | 59 (17.2) |
| Jan 2022 – Nov 2022 | 143 | 47 (32.9) | 21 (14.7) | 40 (28.0)** | 35 (24.5) |
|  |  |  |  |  |  |
|  |  | **“Do you feel that people get comparable health care through telehealth as they do for in-person visits?”** | | | |
|  |  | **Yes, I think the care is comparable** | **No, but telehealth is a good option for initial consultations and/or basic care** | **No, telehealth care will never match the quality of an in-person visit** | **Don’t know or unsure** |
| **Characteristic** | **N** | **n (%)** | **n (%)** | **n (%)** | **n (%)** |
| **All participants** | 487 | 49 (10.1) | 273 (56.1) | 145 (29.8) | 18 (4.7) |
| **Age** |  |  |  |  |  |
| <65 years | 72 | 10 (13.9) | 40 (55.6) | 20 (27.8) | 2 (2.8) |
| ≥65 years | 415 | 39 (9.4) | 233 (56.1) | 125 (30.1) | 16 (3.9) |
| **Race** |  |  |  |  |  |
| Black | 122 | 18 (14.8) | 56 (45.9) | 46 (37.7) | 2 (19.7) |
| White | 351 | 30 (8.5) | 208 (59.3)* | 95 (27.1)* | 16 (15.1) |
| Other | 14 | 1 (7.1) | 9 (64.3) | 4 (28.6) | 0 (0.0) |
| **Education** |  |  |  |  |  |
| ≤high school | 140 | 13 (9.3) | 66 (47.1) | 59 (42.1) | 1 (0.7) |
| Any college | 347 | 36 (10.4) | 207 (59.7)* | 86 (24.8)*** | 17 (4.9) |
| **Household income** |  |  |  |  |  |
| ≤$40,000 | 163 | 15 (9.2) | 78 (47.9) | 66 (40.5) | 3 (1.8) |
| >$40,000 | 313 | 34 (10.9) | 188 (60.1)* | 76 (24.3)*** | 14 (4.5) |
| **Rural-urban residence^a^** |  |  |  |  |  |
| Rural | 224 | 17 (7.6) | 124 (55.4) | 75 (33.5) | 8 (3.6) |
| Urban/Mixed | 261 | 32 (12.3) | 148 (56.7) | 70 (26.8) | 10 (3.8) |
| **Time of survey** |  |  |  |  |  |
| Dec 2020 – Dec 2021 | 344 | 37 (10.8) | 195 (56.7) | 96 (27.9) | 15 (4.4) |
| Jan 2022 – Nov 2022 | 143 | 12 (8.4) | 78 (54.5) | 49 (34.3) | 3 (2.1) |
|  |  |  |  |  |  |
|  |  | **“Has the COVID-19 pandemic changed your view of telehealth?”** | | | |
|  |  | **More likely to use telehealth since the COVID-19 pandemic** | **Have the same opinion compared to before the pandemic** | **Less likely to use telehealth since the COVID-19 pandemic** | **Don’t know or unsure** |
| **Characteristic** | **N** | **n (%)** | **n (%)** | **n (%)** | **n (%)** |
| **All participants** | 487 | 80 (16.4) | 342 (70.2) | 60 (12.3) | 3 (0.6) |
| **Age** |  |  |  |  |  |
| <65 years | 72 | 16 (22.2) | 53 (73.6) | 3 (4.2) | 0 (0.0) |
| ≥65 years | 415 | 64 (15.4) | 289 (69.6) | 57 (13.7)* | 3 (0.7) |
| **Race** |  |  |  |  |  |
| Black | 122 | 24 (19.7) | 78 (63.9) | 19 (15.6) | 1 (0.8) |
| White | 351 | 53 (15.1) | 256 (72.9) | 38 (10.8) | 2 (0.6) |
| Other | 14 | 3 (21.4) | 8 (57.1) | 3 (21.4) | 0 (0.0) |
| **Education** |  |  |  |  |  |
| ≤high school | 140 | 11 (7.9) | 95 (67.9) | 33 (23.6) | 0 (0.0) |
| Any college | 347 | 69 (19.9)** | 247 (71.2) | 27 (7.8)*** | 3 (0.9) |
| **Household income** |  |  |  |  |  |
| ≤$40,000 | 163 | 20 (12.3) | 116 (71.2) | 25 (15.3) | 1 (0.6) |
| >$40,000 | 313 | 59 (18.8) | 218 (69.6) | 33 (10.5) | 2 (0.6) |
| **Rural-urban residence^a^** |  |  |  |  |  |
| Rural | 224 | 29 (12.9) | 165 (73.7) | 29 (12.9) | 1 (0.4) |
| Urban/Mixed | 261 | 51 (19.5) | 176 (67.4) | 31 (11.9) | 2 (0.8) |
| **Time of survey** |  |  |  |  |  |
| Dec 2020 – Dec 2021 | 344 | 55 (16.0) | 241 (70.1) | 44 (12.8) | 3 (0.9) |
| Jan 2022 – Nov 2022 | 143 | 25 (17.5) | 101 (70.6) | 16 (11.2) | 0 (0.0) |

^a^ Participants reported whether they considered themselves to live in a primarily rural, primarily urban or mixed location at the time of the survey.

**p*<0.05

***p*<0.01

****p*<0.001
